# Swept-source optical coherence tomography findings in premature children with a history of retinopathy of prematurity at 5 years of age

**DOI:** 10.5935/0004-2749.20200090

**Published:** 2024-02-11

**Authors:** Gokhan Celik, Murat Gunay, Osman Kizilay

**Affiliations:** 1 Department of Ophthalmology, Zeynep Kamil Maternity and Children’s Diseases Training and Research Hospital, Istanbul, Turkey; 2 Department of Ophthalmology, Trabzon Fatih State Hospital, Trabzon, Turkey

**Keywords:** Retinopathy of prematurity/drug therapy, Tomography, optical coherence, Bevacizumab/therapeutic use, Light coagulation, Infant, newborn, Retinopatia da prematuridade/tratamento farmacológico, Tomografia de coerência óptica, Bevacizumab/uso te rapêutico, Fotocoagulação, Recém-nascido

## Abstract

**Purpose:**

To compare central foveal thickness, retinal nerve fiber layer thickness, and subfoveal choroidal thickness using swept-source optical coherence tomography in premature children with a history of treated retinopathy of prematurity (either with intravitreal bevacizumab or laser photocoagulation) or spontaneously regressed retinopathy of prematurity versus age-matched healthy children at the age of 5 years.

**Methods:**

A total of 79 children were divided into four groups: group 1, children who received intravitreal bevacizumab treatment; group 2, children who received laser photocoagulation treatment; group 3, children who had spontaneously regressed retinopathy of prematurity; and group 4, age matched, full-term healthy children. At the age of 5 years, visual functions and refractive status were assessed. The optical coherence tomography analysis was performed using swept-source optical coherence tomography (DRI-OCT Triton; Topcon, USA).

**Results:**

There were 12 (15.2%), 23 (29.1%), 30 (38%), and 14 (17.7%) children in groups 1, 2, 3, and 4, respectively. Sex distribution was similar between the groups (p=0.420). Best corrected visual acuity was significantly better in group 4 compared with groups 1, 2, and 3 (p=0.035, p=0.001, and p=0.001, respectively). Refractive error results were similar between the groups (p=0.119). Central foveal thickness was significantly higher in group 2 than in group 1 (p=0.023). There were no significant differences observed between the groups in retinal nerve fiber layer thickness and subfoveal choroidal thickness (p>0.05).

**Conclusions:**

Visual functional outcomes were better in term-born healthy children compared with those noted in children with a history of treated retinopathy of prematurity and spontaneously regressed retinopathy of prematurity. Laser treatment exerted a signifi cant effect on central foveal thickness in premature children at the age of 5 years, as revealed by swept-source optical coherence tomography.

## Abstract (pt)

**Objetivo:**

Comparar a espessura central foveal, a da camada de fibras nervosas da retina e a da coróide subfoveal através da tomografia de coerência óptica *swept-source* em crianças de 5 anos de idade com história de retinopatia da prematuridade (RP) tratada com bevacizumabe intravítreo, ou com fo tocoagulação a *laser*, com crianças em regressão espontânea da retinopatia da prematuridade, e com crianças saudáveis da mes ma idade.

**Métodos:**

Um total de 79 crianças foi dividido em quatro grupos. Grupo 1: crianças que receberam tratamento com bevacizumabe intravítreo. Grupo 2: crianças que foram tratadas com fotocoagulação a *laser*. Grupo 3: crianças que tiveram regressão espontânea da retinopatia da prematuridade . Grupo 4: crianças da mesma idade saudáveis e nascidas a termo. As funções visuais e o *status* refrativo foram avaliados aos 5 anos de idade. A análise de tomografia de coerência óptica foi feita por um dispositivo do tipo *swept-source* (DRI-OCT Triton; Topcon, EUA).

**Resultados:**

Haviam 12 crianças (15,2%) no grupo 1, 23 crianças (29,1%) no grupo 2, 30 crianças (38%) no grupo 3 e 14 crianças (17,7%) no grupo 4. A distribuição por sexo foi semelhante em todos os grupos (p=0,420). A acuidade visual com a melhor correção mostrou-se significativamente maior no grupo 4 em comparação com os grupos 1, 2 e 3 (respectivamente, p=0,035, p=0,001 e p=0,001). Os resultados dos erros de refração foram semelhantes em todos os grupos (p=0,119). A espessura foveal central mostrou-se significativamente maior no grupo 2 do que no grupo 1 (p=0,023). Não foram observadas diferenças significativas entre os grupos quanto à espessura da camada de fibras nervosas da retina e à espessura da coroide subfoveal (p>0,05).

**Conclusões:**

Os desfechos visuais funcionais foram melhores nas crianças saudáveis nascidas a termo, em comparação com aqueles observados nas crianças com história de retinopatia da prematuridade tratada ou com regressão espontânea. O tratamento com *laser* teve um efeito significativo na espessura foveal central em crianças de 5 anos de idade, nascidas prematuras, como revelado pela tomografia de coerência óptica *swept-source*.

## INTRODUCTION

Retinopathy of prematurity (ROP) is a proliferative vascular disorder of the developing retina in premature infants. Abnormal production of vascular endothelial growth factor from avascular retina plays a great role in the development of ROP^(1)^. Laser photocoagulation (LPC) has been successfully used to treat the disease and has become the primary treatment modality of ROP^(2)^. Following the introduction of intravitreal anti-vascular endothelial growth factor injections for the treatment of severe cases of ROP, more favorable anatomic and functional outcomes have been observed compared with those obtained through LPC^(3)^. The Bevacizumab Eliminates the Angiogenic Threat of ROP (BEAT-ROP) study, a prospective, controlled, randomized, and multicenter trial, has shown that monotherapy with intravitreal bevacizumab (IVB) has a significant treatment benefit for the infants, especially those with Zone I ROP, compared with LPC^(4)^.

Studies using spectral-domain optical coherence tomography (SD-OCT) have demonstrated significant retinal changes in preterm children with and without ROP^(5^-^7)^. Swept-source OCT (SS-OCT) is a relatively new OCT modality with a longer wavelength than standard SD-OCT. This allows deeper penetration into and better visualization of ocular structures^(8)^. The number of studies evaluating the retinal status with SS-OCT in children with ROP is limited. A study has found that lower choroidal thickness (CT) is associated with the degree of ROP severity in SS-OCT analysis^(9)^.

In the present study, we used SS-OCT to examine central foveal thickness (CFT), retinal nerve fiber layer thickness (RNFLT), and subfoveal CT (SFCT). Subsequently, we compared these parameters in children with a history of treated ROP (either with IVB or LPC) or spontaneously regressed ROP versus age-matched full-term children at the age of 5 years.

## METHODS

This study was approved by the local ethics committee of Zeynep Kamil Maternity and Children’s Diseases Training and Research Hospital and performed in accordance with the ethical standards outlined in the Declaration of Helsinki. The study was performed in Zeynep Kamil Maternity and Children’s Diseases Training and Research Hospital. Informed consent was provided by the parents or guardians of all children prior to inclusion in the study. Patients with a history of neurologic disorder or any systemic abnormality which could prevent cooperation in the tests were excluded from the study. Children with any ocular pathology other than ROP were also excluded. Patients with unfavorable anatomic outcome and cicatricial abnormalities of ROP, including macular fold, macular dragging and retinal detachment, were excluded. All children in the study were born between 2011 and 2013. Children were classified into four groups: children treated with IVB (group 1); children treated with LPC (group 2); children with a history of spontaneously regressed ROP (group 3); and age matched, full-term healthy children (group 4).

All premature children in the study underwent standard ROP screening examinations at 4 weeks following birth based on international guidelines^(10)^. Children who met the criteria for treatment received either IVB or LPC. Treatment indications were based on established guidelines^(2)^. The parents were also informed regarding the lower efficacy of treatment with LPC in posterior ROP and possible side effects (e.g., prevention of peripheral retinal vascularization and higher refractive outcomes) compared with IVB^(4)^. The parents subsequently decided the treatment (i.e., IVB or LPC) to be administered. Decisions to treat infants with IVB were made after informing the patients and/or guardians regarding possible treatment effects and systemic concerns associated with bevacizumab.

All children underwent ophthalmologic examination at the age of 5 years. Best corrected visual acuity (BCVA) was measured in all childern. Refractive errors were identified 45 min after applying 1% cyclopentolate (administered in two instillations separated by an interval of 10 min) using a handheld autorefractometer (HandyRef-K Autorefractometer; Nidek, Gamagori, Japan). Retinoscopy was subsequently performed to refine the refractive result.

### Optical coherence tomography analysis

**Figure 1:**
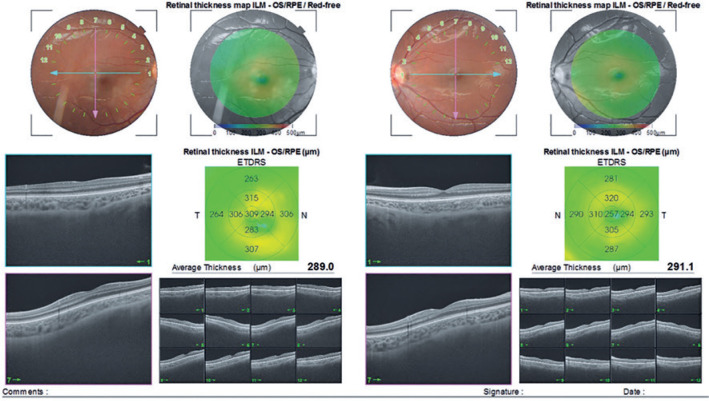
A representative swept-source optical coherence tomography image of a child who received intravitreal bevacizumab.

The SS-OCT (Deep Range Imaging OCT; Topcon, Tokyo, Japan) analysis was performed in all children. The device uses a wavelength-sweeping laser with a center wavelength of 1,050 nm and a tuning range of approximately 100 nm. The thicknesses of the retinal layers were manually quantified from the OCT images in a central single scan. All eyes in the study were examined using the wide-angle (12×9 mm) scan setting centered on the posterior pole. The CFT, RNFLT, and SFCT were obtained from the built-in software and automatically calculated in super pixel grids. The CFT was measured between internal limiting membrane and retinal pigment epithelium. Numeric averages of the CFT measurements were calculated for each of the nine map fields defined by the Early Treatment Diabetic Retinopathy Study. The RNFLT was measured between the internal limiting membrane and ganglion cell layer. A circumpapillary circle (diameter: 3.4 mm) was automatically placed and centered on optic disc, and the RNFLT along the circle was determined after segmentation. It was divided into a grid with four equal sectors for superior, temporal, inferior, and nasal RNFLT. The average RNFLT measurements were used for the statistical analysis. The CT profile was identified by manually measuring the SFCT from the posterior edge of the retina pigment epithelium to the choroidoscleral junction as previously described^(11)^. Figure 1 represents a swept-source optical coherence tomography image of a child who received IVB.

### Statistical analysis

The IBM SPSS Statistics 22 (IBM SPSS, Turkey) software was used for the statistical analysis. Descriptive statistical data are presented as mean and standard deviation. The Shapiro-Wilk test was used to assess the distribution of the data. One-way analysis of variance and the Kruskal-Wallis test were used to compare the groups according to the distribution of the data. We utilized the Tukey’s honestly significant difference, Tamhane’s T2, and Mann-Whitney U tests to identify the group with differences. Student’s t-test was used to compare the normally distributed data between the groups. Qualitative data were compared using the chi-squared and Fisher-FreemanHalton exact tests. The association between the normally distributed parameters were evaluated with Pearson correlation analysis. P-values <0.05 denoted statistical significance.

## RESULTS

A total of 79 children were enrolled in the study. There were 12 children (15.2%), including seven girls (58.3%) and five boys (41.7%), in group 1; 23 children (29.1%), including eight girls (34.8%) and 15 boys (65.2%), in group 2; 30 children (38%), including 13 girls (43.3%) and 17 boys (56.7%), in group 3; and 14 children (17.7%), including four girls (28.6%) and 10 boys (71.4%), in group 4. Sex distribution was similar between the groups (p=0.420). All infants receiving IVB or LPC had stage 3 ROP during the interventions. In group 1, five infants had zone 1 ROP and seven infants had zone 2 ROP; in group 2, all infants had zone 2 ROP. In group 3, 16 infants (53.3%) showed regressed ROP from stage 1 and 14 infants (46.7%) had regressed ROP from stage 2. Treatment was applied at postmenstrual ages of 34.4 ± 2.5 and 37.8 ± 2.6 weeks in group 1 and group 2, respectively. The BCVA showed a significant difference between the groups. BCVA was significantly better in group 4 compared with groups 1, 2, and 3 (p=0.035, p=0.001, and p=0.001, respectively). Moreover, BCVA was significantly better in group 3 compared with groups 1 and 2 (p=0.001, p=and 0.026, respectively). The refractive status did not significantly differ between the groups (p=0.119). Demographic and clinical characteristics of the children, including gestational age (GA), birth weight (BW), sex, BCVA, and refractive error results, are summarized in table 1.

**Table 1:** Demographic and clinical characteristics of the children included in the study

Characteristic	Group 1 (N=12)	Group 2 (N=23)	Group 3 (N=30)	Group 4 (N=14)	p-value
GA at birth, weeks (mean±SD)	27.83±3.27	28.96±2.03	31.3±1.82	39±1.3	<0.001^a^
BW, g (mean±SD)	979.17±348.77	1,267.17±352.9	1,598.17±408.67	3,193.21±290.59	<0.001^a^
Sex, male/female	7 (58.3%)/	8 (34.8(%)/	13 (43.3%)/	4 (28.6%)/	0.420
	5 (41.7%)	15 (65.2%)	17 (56.7%)	10 (71.4%)	
BCVA, logMAR (mean±SD)	0.18±0.22	0.14±0.13	0.04±0.09	0±0	<0.001^b^
Refractive error, SE (mean±SD)	0.04±1.52	-0.51±3.75	1.04±1.42	0.32±0.85	0.119

a = One-way analysis of variance test;

b = Kruskal-Wallis test;

a,b = Statistically significant value.

GA= gestational age; BW= birth weight; BCVA= best corrected visual acuity; SE= spherical equivalent; SD= standard deviation.

In the SS-OCT analysis, CFT, RNFLT, and SFCT did not significantly differ between boys and girls (p=0.334, p=0.614, and p=0.503, respectively). These results are summarized in table 2. There was a significant difference in CFT between the groups (p=0.04). The CFT was significantly higher in group 2 than in group 1 (p=0.023). There were no other significant differences in CFT observed between the other groups (p>0.05). Also, there was no significant difference observed between the groups in terms of the RNFLT and SFCT measurements (p>0.05). The SS-OCT results are shown in table 3.

**Table 2:** Distribution of SS-OCT parameters among girls and boys in the study

	Girls	Boys	p-value
CFT, µm	279.18±17.3	283.23±18.72	0.334
RNFLT, µm	110.63±13.02	109.02±14.36	0.614
SFCT, µm	280.88±55.27	290.64±68.07	0.503

Values are presented as the mean±SD.

SS-OCT= swept-source optical coherence tomography; CFT= central foveal thickness; RNFLT= retinal nerve fiber layer thickness; SFCT= subfoveal choroidal thickness; SD= standard deviation.

**Table 3:** Distribution of SS-OCT parameters in the study

	Group 1	Group 2		Group 3		Group 4	p-value
CFT, µm	272.5 ± 12.13	288.61 ± 20.77		281.85 ± 13.7		283.64 ± 10.22	0.040^a^
RNFLT, µm	105.33 ± 16.22	112.04 ± 14.97		108.23 ± 13.12		112.57 ± 10.49	0.426
SFCT, µm	261.17 ± 49.63	285.91 ± 77.31		287.5 ± 60.91		308.07 ± 47.42	0.313

Values are presented as the mean±SD.

^a^= One-way analysis of variance test;

a = Statistically significant value.

CFT= central foveal thickness; RNFLT= retinal nerve fiber layer thickness; SFCT= subfoveal choroidal thickness; SD= standard deviation.

After performing a correlation analysis, it was found that the spherical equivalent (SE) values and RNFLT were positively correlated (r=0.225; p=0.047). Furthermore, higher GA and BW were associated with higher SFCT measurements (r=0.243, p=0.031; r=0.265, p=0.018, respectively). These results are summarized in table 4.

**Table 4:** Correlation results

	CFT, µm	RNFLT, µm	SFCT, µm
BCVA, logMAR, (r; p)	-0.216; 0.056	-0.174; 0.124	-0.08; 0.486
Stereoacuity, degrees of arc (r; p)	-0.125; 0.273	0.051; 0.657	0.099; 0.384
GA, weeks (r; p)	-0.121; 0.288	0.101; 0.375	0.243; 0.031^a^
BW, g (r; p)	-0.088; 0.440	0.135; 0.235	0.265; 0.018^a^
SE, D (r; p)	0.200; 0.077	0.225; 0.047^a^	0.281; 0.012^a^

Pearson correlation analysis,

a = Statistically significant value

BCVA= best corrected visual acuity; GA= gestational age; BW= birth weight; CFT= central foveal thickness; RNFLT= retinal nerve fiber layer thickness; SFCT= subfoveal choroidal thickness; SE= spherical equivalent.

## DISCUSSION

In this study, we observed a significantly thicker central fovea in laser-treated children than in those who received treatment with IVB. The RNFLT and SFCT did not show significant changes between the study groups.

It has been shown that prematurity is associated with an increased incidence of unfavorable visual functional outcomes^(12)^. Studies have also demonstrated a damaging effect of ROP on visual acuity in premature children^(13^,^14)^. In our study, we found consistent findings with the literature that full-term healthy children had significantly better BCVA outcomes than premature children. Our results also showed that treatment of ROP either with IVB or LPC in premature children negatively impacts visual outcomes. This was revealed by better BCVA values recorded in children with a history of spontaneously regressed ROP than in those who received treatment for ROP.

The refractive status has been reported to be dissimilar between prematurely born and term-born children. It has been shown that premature children have a higher incidence of higher refractive errors^(15)^. The magnitude of refractive progression is significantly higher in patients treated for ROP compared with those who had spontaneously regressed ROP^(16)^. Our findings regarding the refractive status were contradictory to those reported in the literature. In our study, we did not detect any significant difference in refractive error development between premature and healthy term infants. In our opinion, these results may be attributed to the relatively lower number of children in each group, and predominantly zone 2 involvement in both IVB and LPC treatment groups.

Studies using OCT have reported several changes in the posterior pole of premature children. Regarding the CFT, Wu et al.^(17)^ found a higher mean foveal thickness in premature children with a history of laser ablation than in those with regressed ROP and full-term healthy children (mean age: 9 years). Akerblom et al.^(18)^ have also identified significantly greater CFT in prematurely born children compared with term-born children. The development of a thicker fovea in preterm children remains controversial. Studies have shown abnormal development of the macula in preterm infants. It has been demonstrated that existence of macular edema in the premature period, as well as the presence of retinal layers other than outer nuclear layer in the central part of the fovea may affect the CFT in premature children. Furthermore, lateral displacement of inner retinal cells during foveal development in premature infants has been proposed to result in a thicker retina^(17^,^18)^. Our results showed that laser-treated children had significantly higher CFT than IVB-treated children. There were no other significant differences in CFT observed in the other groups. In a recent study, investigators analyzed early foveal development following treatments with IVB and laser. They found that eyes treated with IVB had a higher rate of outer retinal thickening versus untreated eyes^(19)^. In another study, it was shown that the retinal layers in the macula remained thicker in laserand laser+IVB-treated children versus children who recei ved treatment with IVB as monotherapy^(20)^. Several assumptions have been made regarding the thicker central fovea observed in laser-treated children. Compared with IVB, LPC destroys the peripheral avascular retina. This leads to blockage of peripheral migration and reorganization of the inner retinal cells, resulting in a thicker fovea. Furthermore, it has been postulated that LPC-induced inflammation may have an impact on cellular migrational arrest at a certain point during foveal deve lopment. In contrast, treatment with IVB may allow inner retinal cells to continue their migration, thus resulting in normal foveal development^(20)^.

A study evaluated the RNFLT among premature infants and showed that children who had received laser ablation for ROP have lower RNFLT than full-term children and premature children without ROP. The authors assumed that severe ROP may harm ganglion cell axons, reducing the RNFLT^(21)^. Studies have also revealed thinning of the RNFL following the use of panretinal LPC for other retinal vascular disorders, such as diabetic retinopathy^(22)^. Park and Oh^(23)^ found decreased RNFLT in premature children compared with full-term children. Another study observed a relationship between reduced RNFLT and the degree of prematurity irrespective of the presence of ROP^(24)^. In the present study, we did not identify a significant difference in RNFLT between the groups. However, further correlation analysis revealed a significant relationship between decreased RNFLT and lower SE values. Since a thinner RNFL has been associated with myopic refraction^(25)^, this finding is consistent with the literature.

Erol et al.^(5)^ found an association of a thin SFCT with ROP progression, as measured in children aged 36-42 weeks. In another study, it was shown that the SFCT increased with age in premature infants; however, it was thinner compared with that measured in term infants (median age: 34-37 weeks). It has been suggested that oxidative stress in ROP induces choroidal vascular endothelial damage, consequently resulting in a thinner choroid^(26)^. The CT has also been studied in children aged 4-10 years; there was no difference observed in the SFCT between preterm and full-term children^(27)^. In another study, Sayman Muslubas et al.^(28)^ did not report a significant difference in CT between patients with a history of laser treatment, regressed ROP, and full-term children. Using SS-OCT, Bowl et al.^(9)^ revealed a significantly thinner choroid in children with a history of treatment and spontaneously regressed ROP versus term-born healthy children. The authors have also shown a positive dependence of BW on the SFCT, whereas other studies did not find a significant association between the SFCT, GA, and BW^(27^,^29)^. In our study, the SFCT was found to be higher in full-term born children compared with prematurely born children. However, this finding did not reach statistical significance. Moreover, the correlation analysis of this study showed that higher GA and BW were significantly associated with increased SFCT.

In conclusion, the results of the present study showed a significantly higher CFT in laser-treated children. The RNFLT and SFCT did not significantly differ between the groups. In addition, BCVA was better in term-born healthy children versus those with a history of treated ROP and spontaneously regressed ROP. Since OCT measurements can be influenced by the axial length^(30)^, an important limitation of the present study is that the biometric profile of the children was not assessed. This may have had an impact on the SFCT measurements in the present study. Future prospective, large-series studies are warranted to validate the results of the present study and better ascertain the SS-OCT outcomes in children with a history of ROP.
